# Individual differences in blink rate modulate the effect of instrumental control on subsequent Pavlovian responding

**DOI:** 10.1007/s00213-018-5082-6

**Published:** 2018-11-01

**Authors:** Catherine A. Hartley, Cesar A. O. Coelho, Emily Boeke, Franchesca Ramirez, Elizabeth A. Phelps

**Affiliations:** 1 Department of Psychology, New York University, New York, USA; 2 Center for Neural Science, New York University, New York, USA; 3 Departamento de Psicobiologia, Universidade Federal de São Paulo, São Paulo, Brazil; 4 Department of Psychology, Harvard University, Cambridge, USA; 5 Emotional Brain Institute, Nathan Kline Institute for Psychiatric Research, Orangeburg, USA

**Keywords:** Pavlovian learning, Conditioned responses, Dopamine, Active avoidance

## Abstract

**Rationale:**

Pavlovian conditioned responses to cues that signal threat are rapidly acquired and tend to persist over time. However, recent research suggests that the ability to actively avoid or exert control over an anticipated threat can diminish the subsequent expression of Pavlovian responses. Studies in animal models suggest that active avoidance behavior and its consequences may be mediated by dopaminergic function. In the present study, we sought to replicate the finding that active control over threat can attenuate subsequent Pavlovian responding in humans and conducted exploratory analyses testing whether individual differences in blink rate, a putative index of dopaminergic function, might modulate this effect.

**Methods:**

Participants underwent Pavlovian aversive conditioning, followed immediately by one of two conditions. In the active avoidance condition, participants had the opportunity to actively prevent the occurrence of an anticipated shock, whereas in a yoked extinction condition, participants passively observed the cessation of shocks, but with no ability to influence their occurrence. The following day, the conditioned stimuli were presented without shock, but both groups of participants had no opportunity to employ active instrumental responses. Blink rate was measured throughout the task, and skin conductance responses served as our index of Pavlovian conditioned responding.

**Results:**

Consistent with our previous findings, we observed that the group that could actively avoid the shock on day 1 exhibited attenuated recovery of Pavlovian conditioned responses. Further, we found that individuals in the active avoidance group with higher blink rates exhibited a more robust attenuation of spontaneous recovery.

**Conclusion:**

This finding suggests that individual variation in dopaminergic function may modulate the efficacy with which active avoidance strategies can attenuate reactive Pavlovian responses.

**Electronic supplementary material:**

The online version of this article (10.1007/s00213-018-5082-6) contains supplementary material, which is available to authorized users.

## Introduction

Through Pavlovian learning, cues that signal the potential for danger can elicit reactive defensive responses, typically characterized by the inhibition of ongoing behavior and heightened physiological arousal (Bolles 1970; Pavlov 1927). Conditioned defensive reactions provide an evolutionarily prescribed response strategy that can be deployed automatically in threatening situations without deliberation. While Pavlovian learning is persistent, its expression can be altered in accordance with changes in the predictive validity of threat cues. When a previous predictor of threat is no longer followed by aversive outcomes, expression of conditioned Pavlovian responses to the cue typically decreases—a process referred to as extinction learning. However, the memory for the prior predictive relationship appears to be retained, as conditioned responses to the previously threat-predictive stimulus often reemerge following the passage of time (“spontaneous recovery”), changes in context (“renewal”), or exposure to aversive outcomes (“reinstatement”), all of which may render the probability of threat or safety more ambiguous (Bouton 2004).

Although Pavlovian learning equips an organism with a “default” behavioral response strategy, there are other potential ways to respond to environmental threats (LeDoux and Daw 2018). In many cases, when faced with potential negative outcomes, we may try to discover adaptive behaviors that can proactively prevent these aversive events or mitigate their impact. In controllable situations, where one’s actions can effectively disarm threats, such active instrumental responses may bring about better outcomes than Pavlovian conditioned responses. In turn, these reinforcing outcomes may increase the propensity to respond proactively to threats that are encountered in the future. In this manner, situations that afford the opportunity to control a stressor may promote recruitment of active responses over reactive Pavlovian responses when facing novel or ambiguous behavioral challenges (Moscarello and Hartley 2017). By learning that one can cope effectively with threats through action, the need to rely on stimulus-bound conditioned responses may be diminished.

Convergent experimental findings have provided support for this model. In humans and rodents, the ability to learn active responses to terminate or avoid an aversive stimulus attenuates the reemergence of Pavlovian responding (Baratta et al. 2007; Boeke et al. 2017; Cain and LeDoux 2007; Hartley et al. 2014; Kamin et al. 1963) and promotes active responses in subsequent situations where the degree of threat controllability may be ambiguous (Amat et al. 2005; Seligman and Maier 1967). Moreover, work in animal models suggests that the effects of control generalize broadly, promoting proactive responding even in situations that may be qualitatively different in nature than previously encountered controllable stressors (Maier 2015). These findings are consistent with an account in which individuals use their past experience to derive an a priori belief about the likelihood that they will be able to exert control in novel situations, biasing their behavioral responses accordingly (Huys and Dayan 2009; Lieder et al. 2013; Moscarello and Hartley 2017). Such an account, akin to the longstanding social psychological formulation of an internal locus of control (Rotter 1966), suggests that the controllability of one’s real-world experiences might similarly give rise to a general tendency for an individual to cope proactively or reactively with novel challenges. Such trait-like individual differences in coping strategies have been observed in animal models (Koolhaas et al. 1999); however, there has been little study of such variation or its underlying biological mechanisms in humans.

Recent findings in animal models suggest multiple ways in which the dopaminergic system might modulate the tendency to employ proactive versus reactive coping strategies. Striatal dopamine is essential for the acquisition and expression of active avoidance responses (Darvas et al. 2011; Fibiger et al. 1974; Koob et al. 1984). Dopaminergic neurons projecting to the striatum are thought to reinforce active responses to threat (Menegas et al. 2018), highlighting a central role for dopamine in avoidance learning. Dopamine is also implicated in the consolidation of active avoidance learning (Gozzani and Izquierdo 1976), enabling the modulation of active coping responses at timepoints subsequent to initial learning. Optogenetic facilitation or inhibition of dopamine activity respectively elicits active or passive responding in response to an uncontrollable stressor (Tye et al. 2013). Rats exposed to threats that can be actively avoided exhibit increases in dopamine release in the striatum and concurrent increases in active coping behavior, whereas uncontrollable threat exposure produces a decrease in dopamine release and the expression of passive freezing responses (Oleson et al. 2012). These results suggest that fluctuations in dopamine signaling at multiple timescales are sensitive to past experiences of control and can modulate the learning and expression of active versus reactive threat responses. Collectively, these findings have motivated a proposal that increased dopamine may promote a motivational state in which proactive exploration and discovery of available adaptive actions are facilitated and reactive Pavlovian responses are suppressed (Cabib and Puglisi-Allegra 2012; Lloyd and Dayan 2016).

While dopamine levels cannot directly be measured in humans, a host of studies suggest that the spontaneous rate of eyeblink provides a physiological correlate of central dopaminergic function that is easily measured noninvasively (Jongkees and Colzato 2016). Blink rate is increased by pharmacological dopamine agonists, and reduced by dopamine antagonists (Elsworth et al. 1991; Taylor et al. 1999). Blink rate is decreased in neurological disorders associated with reduced striatal dopamine (Karson et al. 1984). Blink rate has been found to correlate with postmortem measures of striatal dopamine concentration (Taylor et al. 1999), as well as PET measures of D2-like receptor availability (Groman et al. 2014), in the striatum in non-human primates. However, this association between blink rate and D2-like receptor availability has not been consistently replicated in humans (Dang et al. 2017; Sescousse et al. 2018), and the precise mechanism linking dopamine signaling to blink rate remains unclear. To the extent that blink rate can provide an index of dopaminergic function, the previously described findings in animal models suggest that individuals with a higher blink rate might exhibit facilitated avoidance learning and expression and be more likely to favor active instrumental strategies to cope with threats over Pavlovian reactive responses.

Our previous work has shown that the ability to actively avoid a learned threat diminishes the subsequent expression of Pavlovian responses (Boeke et al. 2017; Hartley et al. 2014). In the present study, we sought to replicate this finding and to test whether the effects of controllability might relate to individual differences in blink rate. Participants underwent Pavlovian aversive conditioning (acquisition phase), followed immediately by either an opportunity to actively prevent the occurrence of an anticipated shock (active avoidance phase), or the passive observation of the cessation of shocks (yoked extinction phase). The next day, participants were presented with the conditioned stimuli from the previous day, but with no opportunity to employ active instrumental responses (retrieval phase). Consistent with our previous findings (Boeke et al. 2017; Hartley et al. 2014), we found in this independent sample that the ability to exert instrumental control attenuated the spontaneous recovery of Pavlovian conditioned responses for participants in the active avoidance condition the next day, relative to those in the yoked extinction condition.

Convergent evidence suggests that dopamine modulates active avoidance learning and expression (Lloyd and Dayan 2016) and that blink rate can provide a putative index of individual differences in dopaminergic function (Jongkees and Colzato 2016). Following our analysis of the conditioning data, we conducted secondary exploratory analyses using pupillometry data collected during the task to test whether participants’ blink rates might modulate the observed effects of control on subsequent Pavlovian responding. We had the following three specific hypotheses suggested by observed relationships between dopamine and active coping behavior in animal models. First, based on evidence suggesting a critical role for dopamine in avoidance learning and expression (Darvas et al. 2011; Fibiger et al. 1974; Koob et al. 1984; (Menegas et al. 2018), we hypothesized that blink rate might be associated with better avoidance learning in participants who had the opportunity to prevent shock delivery. Second, based on convergent lines of evidence showing that dopamine is involved in the consolidation of active avoidance learning (Gozzani and Izquierdo 1976) and can facilitate the expression of active over passive behavioral responses (Tye et al. 2013), we hypothesized that individuals with higher blink rates might exhibit stronger consolidation of avoidance learning, and exhibit a greater bias toward active over passive responding, attenuating expression of reactive conditioned responses during the retrieval phase. Finally, based on evidence suggesting that tonic dopamine levels are sensitive to the controllability of threat (Oleson et al. 2012), we hypothesized that participants in the active avoidance condition might exhibit increases in blink rate from the acquisition to avoidance phase, whereas those who underwent yoked extinction would not.

## Material and methods

### Participants

All participants provided informed consent and were paid for their participation. Eighty-five subjects completed the study and were randomly assigned to an active avoidance (AA) or yoked extinction (EXT) condition. Fourteen additional subjects completed day 1 but not day 2 and are therefore not included in any analyses. Six AA subjects were excluded prior to yoking for not successfully learning the avoidance response. Additional subjects were excluded due to a lack of variable skin conductance responses (SCRs) (“non-responders,” *n* = 5), not acquiring a conditioned response (“non-acquirers,” *n* = 14; see the “Psychophysiological assessment” section), or technical problems (*n* = 5). Six EXT participants were excluded because their AA counterparts were found to be non-acquirers or non-responders. One EXT subject was an inadvertently duplicated yoke, and thus was not retained in the final sample. The final sample included 48 subjects, 24 in each group (AA mean age 22.04 ± 4.19 years, 16 females; EXT mean age 22.32 ± 3.27 years, 14 females).

### Procedure

**Fig. 1:**
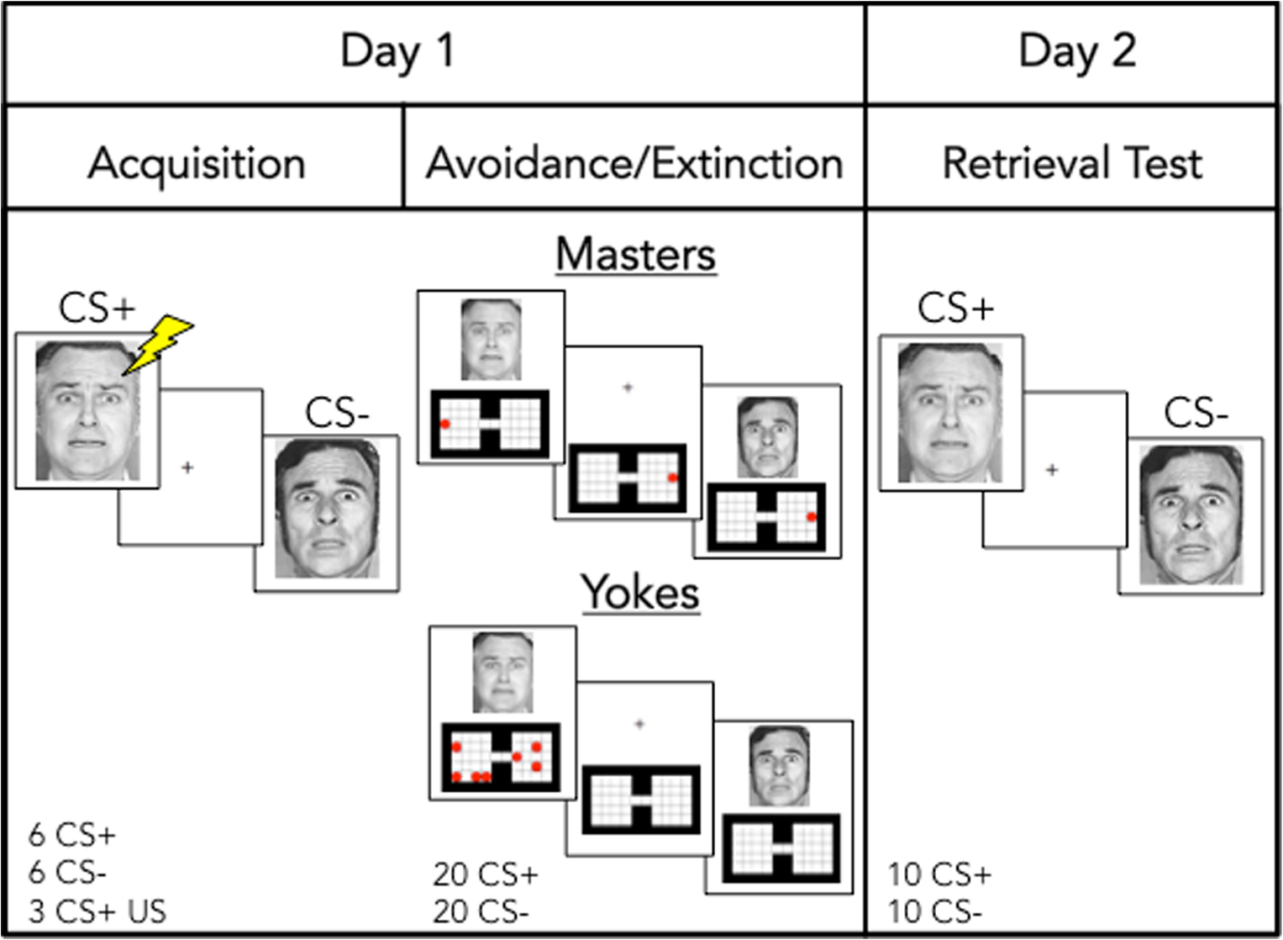
Experimental paradigm. **a** Schematic sequence of events of acquisition and signaled active avoidance (or yoked extinction). **b** Number of each type of CS per session on both days of the experiment

The study consisted of three phases spanning 2 days: acquisition, AA or yoked EXT, and retrieval test (Fig. 1). At the start of day 1, participants calibrated the level of a mild electric shock to a level deemed “uncomfortable, but not painful.” The 200-ms electrical pulse was delivered to the wrist of the right hand using a Grass Technologies stimulator (Warwick, RI). Following calibration, participants were given instructions about the task. AA participants were told that they would view two faces, one of which would sometimes be followed by a shock (the “threat face”) and another that would never be followed by a shock (the “no-threat face”) and that their first task was to learn which face was the threat face. Participants were instructed that midway through the task, a grid would appear below the faces. AA participants were told that they would be able to move a circle within the grid using the arrow keys, and that during the threat face presentation, they could try to learn an action that would prevent the shock occurrence. They were not told explicitly what this action was. EXT participants were told that a number of circles would appear in the grid on each trial, and that they should make a number of clicks on a handheld analog clicker device that matched the number of circles in the grid on that trial. They were told that they would not be able to influence the experiment or avoid shocks through their button presses.

After receiving instructions, participants underwent a partial-reinforcement discriminative aversive conditioning procedure (acquisition phase) using two fear faces as stimuli (Ekman et al. 1969). One face (CS+) coterminated with a mild electric shock to the wrist (unconditioned stimulus, US) on 50% of the presentations, and the other face (CS−) was never paired with the shock. Acquisition included six presentations of each CS, of which three CS+ presentations were paired with the shock. Each CS was presented for 6 s with an inter-trial interval (ITI) of 6 s.

Immediately after the acquisition phase, active avoidance participants underwent a signaled AA task (Boeke et al. 2017). During the AA session, an image with two 5 × 4 cell grids bridged by a 1 × 2 cell “tunnel,” resembling two connected chambers (Fig. 1), was present on the screen, and CS images were presented above the grid image. Moving the circle from one grid chamber to the other (crossing the 1 × 2 cell tunnel) during CS+ presentation would prevent shock delivery at the end of the trial. The AA session consisted of 20 CS+ and 20 CS− presentations. Each CS presentation was 8 s long with an 8-s ITI. The number of moves (key presses) in each trial was recorded.

To compare the effects of active avoidance and extinction learning, a second group (EXT) underwent a yoked extinction protocol in which each participant experienced the identical number of shocks as their yoked AA “master” participant, but had no control over shock occurrence. To ensure that EXT participants had no illusion of instrumental control (Hartley et al. 2014), they were not given opportunity to move the dot. Instead, on each trial, the grid below the CS contained a set of fixed dots equal to the number of movements made by their AA counterpart. The EXT participants’ task was to press a handheld analog clicker device as many times as the number of dots in the grid.

The instrumental learning performance of each AA participant determined the number of unreinforced trials EXT participants experienced. To ensure both successful avoidance learning in AA participants, and sufficient unreinforced trials for EXT participants extinguish, AA participants who avoided shock on less than 12 trials (60%) were considered “poor learners” and excluded from the analysis. This exclusion criterion was defined in advance of data collection based on the assumption that 12 unreinforced trials (twice the number of acquisition CS+ trials) would likely be sufficient to produce extinction learning (Coelho et al. 2015; Dunsmoor et al. 2015). We required that at least ten of these 12 avoided trials were experienced contiguously at the end of extinction. Nine AA participants received shocks within the last ten trials, despite having successfully avoided the shock on previous trials. Because shocking EXT participants midway through their yoked extinction phase might compromise their extinction learning, the timing of shock administration for these trials was shifted earlier in time for their nine EXT counterparts to the first eight trials, ensuring that all EXT participants experienced at least ten contiguous unreinforced CS+ presentations during extinction. As this temporal shift violates a strict yoking of EXT and AA participants, all analyses reported here were also repeated in a “strict yoke” sub-sample of the remaining 15 yoked AA-EXT pairs, who experienced identical timing of shocks (see Supplemental Information).

On day 2, 24 h later, the shock electrodes were reattached to the participants and they were informed that the shock level would be set to the same level as day 1. All participants underwent a retrieval test phase consisting of ten unreinforced CS+ and ten CS− presentations. Each CS was 6 s long (6-s ITI). Critically, the grid was not presented in this session.

The faces used in the study were counterbalanced across subjects as CS+ and CS− and stimuli were presented in one of two fixed pseudorandom orders (also counterbalanced) in which no stimulus was presented more than three times in a row.

### Psychophysiological assessment

Skin conductance data were sampled at 200 Hz from the hypothenar eminence of the left palmar surface using pre-gelled snap electrodes connected to an MP-100 BIOPAC system (BIOPAC Systems, Goleta, CA). The data were low-pass filtered (3 Hz) using AcqKnowledge software (BIOPAC Systems), and phasic SCRs were assessed in each trial by taking the base-to-peak difference of the largest ascendant deflection beginning between 0.5 s after the onset of each stimulus (CS and shock) and 0.5 s after offset. Responses lower than 0.01 μs were scored as zero. Raw SCR data was square root transformed to normalize the distribution and divided by the mean unconditioned response to the shocks given during acquisition. The SCR values were averaged in blocks of two trials, and our analysis focused on the first (early) or last (late) block of each session. The conditioned response (CR) was defined as the SCR difference score [CS+ minus CS−] in each block. As active avoidance and extinction learning are predicated on successful initial conditioning, participants who did not show evidence of conditioning in acquisition were excluded from all reported analyses. Successful conditioning was defined as a mean CS+ minus CS− differential SCR across the last three acquisition trials greater than 0.05.

Eye tracking data were collected with an EyeLink 1000 system (SR Research, Kanata, Ontario, Canada) at a sampling rate of 250 Hz. Four subjects’ data were unintentionally collected at a higher sampling rate and were downsampled to match the sampling rate of the other subjects. Blink rate was computed during each phase of the experiment (the 144-s acquisition phase, the 640-s avoidance phase, and the 240-s retrieval test phase).

Blink rate was derived via an automated algorithm in MATLAB from eye tracking data using the pupil area signal. As some individuals do not fully close the eyes during blinks (Jiang et al. 2013), we used an individualized area change threshold to identify blinks. For each subject, we calculated the mean and standard deviation of pupil area signal, excluding timepoints when the area value was zero. We identified candidate eye closures as times when the pupil area value was lower than or equal to the area threshold (one standard deviation below the mean of all non-zero data points) and the area decrease from the previous to the current timepoint was greater than the area change threshold (a change of at least .06 * 1 SD). Next, we identified timepoints where the eye was likely open with these criteria: area value greater than the area threshold, and difference between current timepoint and previous one was less than the area change threshold. For each candidate eye closure, we marked the temporally closest timepoint after that closure where the eye was marked as open as the end timepoint for the blink. We discarded any candidate eye closures that occurred between the first candidate eye closure and the eye opening timepoint. We then discard any blinks with duration less than 100 ms or greater than 500 ms. To calculate blink rate, the total number of blinks per phase was divided by the duration, in minutes, of the phase, excluding any timepoints when signal was lost for > 500 ms. While some studies define as blinks any continuous periods where the pupil area is zero that last for a similar specific duration (Aarts et al. 2012; Pas et al. 2014; Peckham and Johnson 2016), the pupil may not be fully occluded during blinks, which motivated our more complex algorithm. However, for completeness, we investigated the correlation between these two methods of identifying blinks. We found that blink rates determined by periods where the area was zero correlated with the blink rates observed using our algorithm (rho = .86, *p* < 10e-12).

Three subjects were excluded from analyses because > 20% of their eyeblink data from acquisition was unscoreable due to signal loss. Four subjects’ eye tracking data was not saved due to a technical error. Data from the remaining 20 AA and 21 EXT subjects are analyzed here. In analyses examining whether groups’ eyeblink rates differed in the AA/EXT or retrieval phases, one additional AA subject and one additional EXT subject are excluded because > 20% of their eyeblink data during AA or retrieval, respectively, was not scoreable due to signal loss.

### Self-report measures

After completing the retrieval test, the participants completed a post-experimental questionnaire including one item assessing their perception of control over the shock during the AA or yoked EXT phase in a five-point forced choice scale (1 = “not at all,” 5 = “very confident”). They also completed the State-Trait Anxiety Inventory (STAI; (Spielberger et al. 1970)), the Internal Control Index (ICI; (Duttweiler 1984), a psychological measure indexing the degree to which one believes that they can control salient everyday life events, and the Barratt Impulsiveness Scale (BIS-11a; (Patton and Stanford 1995)), a measure of impulsivity. ICI data from three AA participants, BIS data from one EXT participant, and the post-experimental questionnaire from one EXT participant were lost due to technical errors or not collected.

## Results

### SCR results

**Fig. 2:**
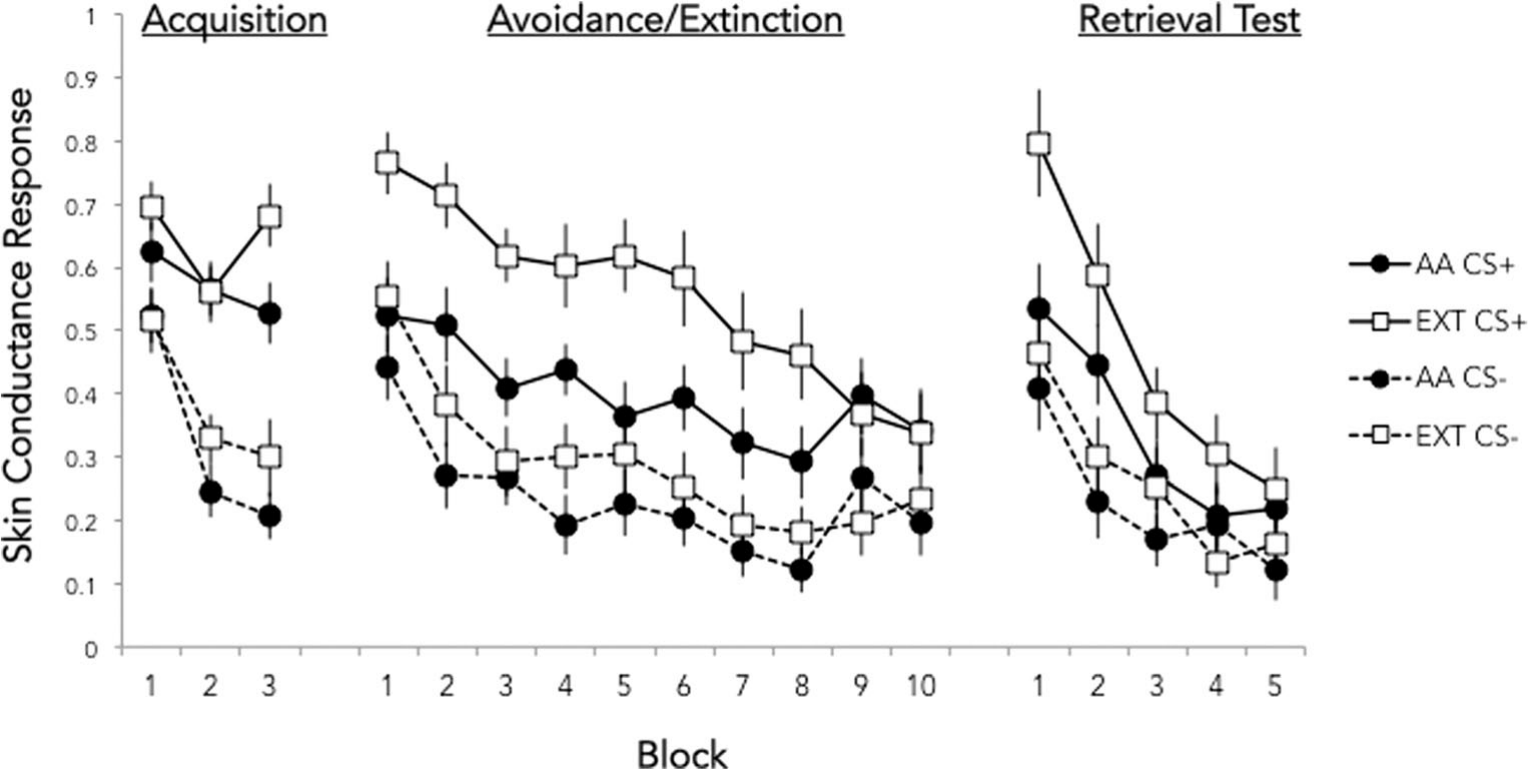
Mean (± standard error) skin conductance response in blocks of 2 trials for each CS and session of both active avoidance (AA) and yoked extinction (EXT) groups (*n* = 24 each)

**Fig. 3:**
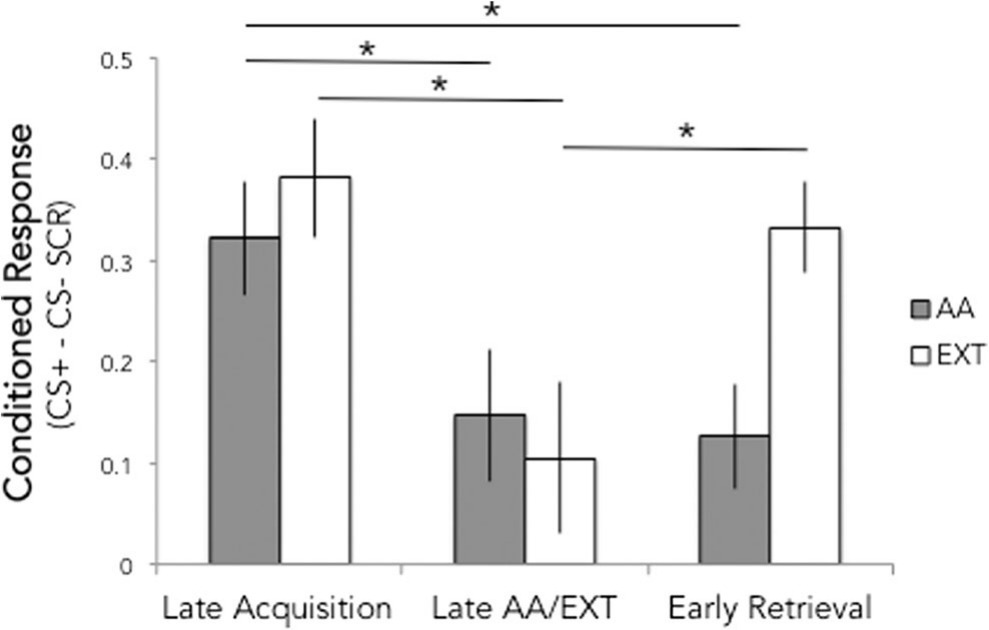
Effect of signaled active avoidance (AA) and yoked extinction (EXT) on conditioned response at timepoints of interest. Mean differential skin conductance response (CS+ minus CS−) during late acquisition, late active avoidance/extinction, and early retrieval for the AA and EXT groups. Both groups exhibited a reduced CR in late AA/EXT, but only the EXT group showed an increase in early retrieval on day 2 compared to late extinction on day 1. CRs in the AA group, but not the EXT group, were lower on day 2 compared to late acquisition. *p < 0.05

Figure 2 depicts the mean SCR to the CS+ and CS− across all blocks of conditioning for each group. We compared the CR (mean CS+ − CS− SCR) across groups during three blocks of interest (late acquisition, late active avoidance (AA)/extinction (EXT), and early retrieval) using a two (group) × three (block) ANOVA (Fig. 3). Where Mauchly’s test indicated that sphericity assumptions were violated, Greenhouse-Geisser correction was applied. Post hoc *t* tests (two-tailed) were conducted to characterize differences between groups and phases.

The ANOVA revealed a main effect of block (*F*(1.71,78.82) = 8.24, *p* = 0.001), no main effect of group (*F*(1,46) = 1.84, *p* = 0.18), and a marginal group × block interaction (*F*(1.71,78.82) = 2.52, *p* = 0.09). Given our strong a priori hypothesis, we followed up on the marginal interaction with two-tailed *t* tests.

Both groups showed a positive differential CR during acquisition (AA *t*(23) = 5.69, *p* < 0.0001; EXT *t*(23) = 7.38, *p* < 0.0001), which did not differ in magnitude between groups (*t*(46) = 0.72, *p* = 0.47). Conditioned responding during late EXT/AA was significantly lower than during late acquisition for both groups (AA *t*(23) = 2.3, *p* = 0.03; EXT *t*(23) = 2.89, *p* = 0.008), and also did not differ between groups (*t*(46) = 0.43, *p* = 0.67).

During early retrieval, the AA group showed lower CR than the EXT group (*t*(46) = 2.99, *p* = 0.004). The CR of the EXT group during early retrieval was greater than zero (*t*(23) = 7.38, *p* < .00001) and higher than during late EXT (*t*(23) = 2.56, *p* = 0.02), whereas the AA group CR during early retrieval was greater than zero (*t*(23) = 2.42, *p* = .02), but did not significantly increase from late AA (*t*(23) = 0.25, *p* = 0.81). We also tested whether conditioned responding of each group during early retrieval was reduced from that of late acquisition. Whereas the AA group exhibited a significantly lower CR during early retrieval compared to late acquisition (*t*(23) = 3.14, *p* = 0.005), no reduction in responding was observed in the EXT group (*t*(23) = 0.83, *p* = 0.41). Collectively, these results suggest that EXT group showed spontaneous recovery of the CR 1 day after extinction, whereas the AA group did not show any evidence of fear recovery after active avoidance learning. These results are consistent with our hypothesis that learning to actively exert control over the aversive stimulus would prevent spontaneous recovery of a Pavlovian CR.

The strict-yoke analysis yielded overall similar results (see Supplemental Information), although the removal of approximately one third of the sample resulted in some loss of statistical power.

### Effects of variable shock level on SCR

Although each yoked EXT participant received the same number of shocks as their AA counterpart, shock intensity was individually calibrated. However, shock intensity did not differ between groups (*t* = 0.54, *p* = 0.59). Moreover, neither number of shocks nor shock intensity correlated with CR during any of the three blocks of interest (besides a trend level negative correlation between number of shocks and CR during AA/EXT (rho = − .24, *p* = .1, all *p*’s > .44). This suggests that our results are unlikely to be driven by differences in shock intensity, and that controllability itself, rather than the number of shocks successfully avoided, appears to underpin group differences in conditioned responding.

### Analysis of blink rate

We first tested whether blink rate during the acquisition phase predicted better avoidance learning performance among AA participants. We observed no relationship between blink rate during acquisition and number of shocks received (a proxy for learning success) in AA participants (rho = − .08, *p* = .7), including both learner and non-learner AA subjects. Nor did we observe a difference in blink rates (*t*(23) = .56, *p* = .58) between AA subjects who met our learning criterion (learners, 21.9 ± 3.3 blinks/min) and those who did not (non-learners, 21.8 ± 7.7 blinks/min). While these results suggest that blink rate does not influence avoidance learning, our study was not ideally suited to test this hypothesis given the relatively low number of non-learners in our study and the coarse measure of number of shocks as a proxy for learning success.

**Fig. 4:**
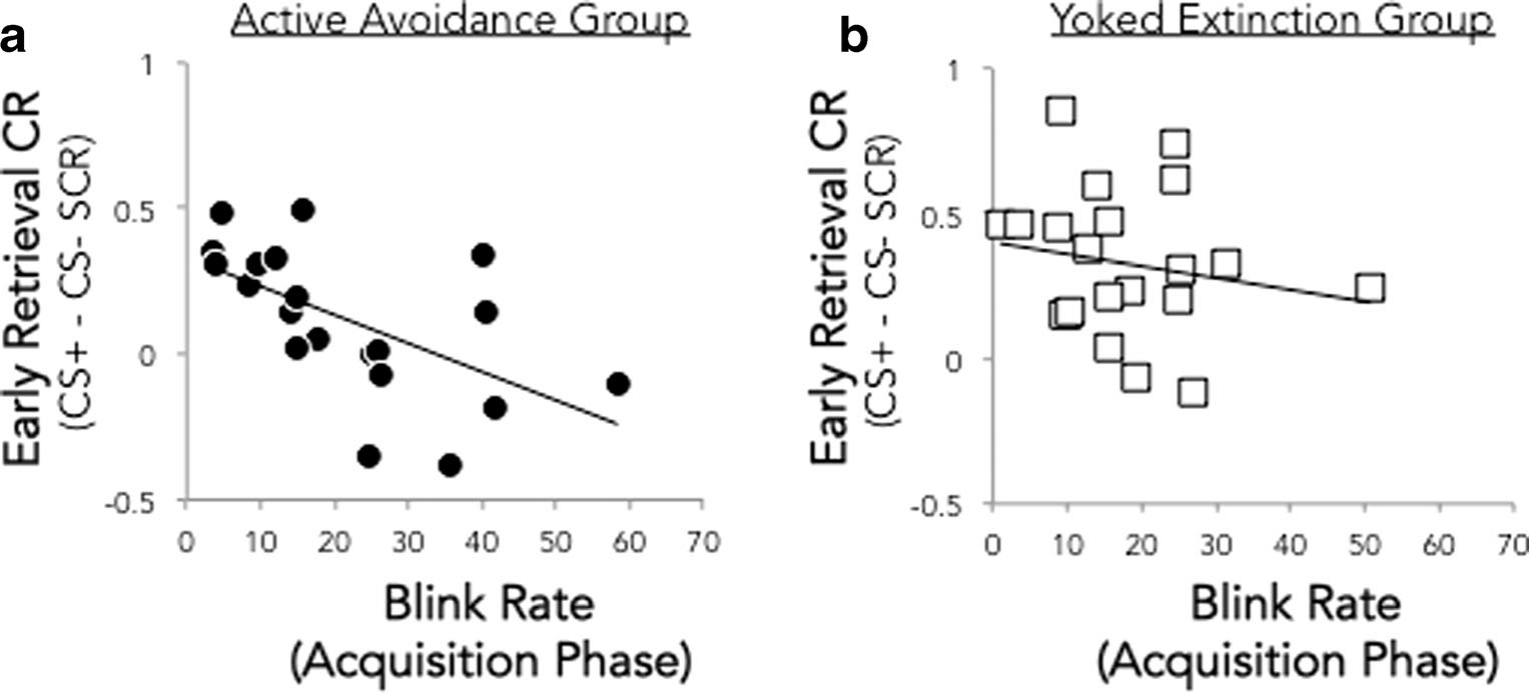
**a**, **b** Higher blink rate during the acquisition phase is associated with lower conditioned response during retrieval in the active avoidance group (*r* = − .65, *p* = .002), but not in the yoked extinction group (*r* = − .21, *p* = .36)

Next, we tested whether blink rate during the acquisition phase predicted the effects of controllability on the recovery of conditioned responding early in the retrieval phase. Specifically, we hypothesized that conditioned responding would be lower in AA participants with higher blink rates. In AA subjects, blink rate was inversely correlated with the CR during the first block of retrieval (rho = − .65, *p* = .002, Fig. 4). The correlation was still significant when a subject with a blink rate greater than three standard deviations from the mean was removed (rho = − .62, *p* = .005). This effect was selective to the CR during the retrieval phase, with acquisition blink rate showing no correlation with the CR during late acquisition (rho = − .17, *p* = .47) or the final block of AA (rho = .06, *p* = .81). This relationship appeared to be specific to the early retrieval timepoint when group differences in CR expression were most pronounced. Acquisition blink rate did not predict CRs during the final block of retrieval (rho = − .31, *p* = .18), when CRs did not significantly differ between groups (*t*(46) = .12, *p* = .91). In EXT subjects, blink rate during acquisition was not associated with differential skin conductance during acquisition or extinction (*p*’s > .15), or the first block of retrieval (rho = − .21, *p* = .36). A Fisher *r* to *z* test of the difference between groups in correlation between acquisition blink rate and early retrieval CR did not reach significance at the two-tailed level (*z* = − 1.66, *p* < .097, two-tailed), but provided support for our specific directional hypothesis.

Since some subjects in each experimental group were omitted from this analysis due to eye tracking data collection problems or poor data quality, the remaining AA and EXT subjects in these analyses were not matched on number of shocks. Thus, we repeated these analyses in the subset of subjects for which both participants in the yoked AA-EXT pair had useable eye tracking data. In this subset of subjects (*N* = 16 per group), we observed the same inverse correlation between AA subjects’ blink rate during acquisition and differential SCR during the first block of retrieval (rho = − .87, *p* = .00001), but not the late acquisition or late AA phases (*p*’s > .41), and no correlation between EXT subjects’ blink rate during acquisition and skin conductance during retrieval (rho = − .26, *p* = .33) or any other phase (acquisition rho = − .44, *p* = 09; EXT rho = − .21, *p* = .42). A Fisher *r* to *z* test confirmed a significant difference between groups in these correlations (*z* = − 2.72, *p* < .0065, two-tailed). Collectively, these results suggest that among subjects assigned to the AA group, who were able to exert control over the aversive stimulus; higher blink rate was associated with a greater attenuation of the Pavlovian CR the following day.

Last, we tested whether blink rates might be sensitive to the ability to deploy active coping strategies and thus, might increase to a greater degree from the acquisition phase to the AA/EXT phase for subjects in the AA condition than for those in the yoked EXT condition. A group (AA/EXT) × phase (acquisition, AA/EXT, retrieval) ANOVA predicting blink rate revealed no main effects of group (*F*(1,37) = .71, *p* = .41) or phase (*F*(1,74) = .24, *p* = .79), and no phase × group interaction (*F*(1,74) = 2.0, *p* = .14) interaction on blink rates. Moreover, blink rate across phases was highly consistent within subjects (for each pair of phases, all rhos > .72, all *p*’s < 1 × 10^−7^). Collectively, these analyses suggest that blink rate was highly stable for each subject and did not exhibit sensitivity to the differential degree of control over aversive reinforcement afforded by the two conditions.

As blink rate across phases was highly consistent for each subject, we additionally examined whether blink rate in other phases of the experiment was related to conditioned responding during early retrieval. In AA subjects, we observed a marginal inverse relationship between blink rate during avoidance and early retrieval CR (rho = − .42, *p* = .07) and a significant inverse correlation between blink rate during retrieval and early retrieval SCR (rho = − .62, *p* = .003). These relationships were not significant in EXT subjects (*p*’s > .16).

### Self-report measures

Participants in the AA group reported a greater subjective perception of control than those in the EXT group (*t*(45) = 4.75, *p* = .00002). Questionnaires scores on the STAI, ICI, and BIS-11 showed no difference between groups (*p* > 0.5 in all cases), as was expected given the random assignment of participants to each group. There was no correlation between subjective perceptions of control or any of the questionnaire measures with conditioned responding during early retrieval (all *p*’s > .11). Blink rate during acquisition did not significantly correlate with questionnaire measures (all *p*’s > .13).

## Discussion

In this study, we found that the ability to proactively prevent the occurrence of an aversive outcome attenuated the subsequent expression of Pavlovian threat responses. Participants who learned to avoid shock exposure by performing a keyboard-based shuttle response when the threat-predictive stimulus was presented did not exhibit spontaneous recovery of Pavlovian threat responses the following day, even though they were no longer able to perform the instrumental response. In contrast, participants who experienced the same cessation of shock, but with no capacity to exert instrumental control (i.e., yoked extinction), showed the typical recovery of threat responses. These results corroborate our previous finding that active control over a conditioned stimulus is more effective than passive extinction learning in diminishing subsequent Pavlovian responding (Boeke et al. 2017). These findings are also consistent with a broader literature suggesting that controllable or uncontrollable experiences may respectively attenuate or facilitate the degree to which an individual expresses Pavlovian reactive responses to subsequent potential threats (Maier 2015; Moscarello and Hartley 2017).

Our results suggest that learning a proactive response to prevent the occurrence of an aversive outcome diminishes subsequent reactive threat responses. However, other studies have found that threat responses reemerge when avoidance behaviors are performed during extinction (Lovibond et al. 2009; Vervliet and Indekeu 2015; Volders et al. 2012). Similarly, studies in clinical samples suggest that the performance of safety behaviors during extinction-based exposure therapy sessions impedes the attenuation of subjective fear (Blakey and Abramowitz 2016). This phenomenon, referred to as “protection from extinction,” is thought to occur because subjects attribute the absence of the negative outcome to the avoidance action, as opposed to learning that the threat-associated stimulus or situation is truly safe. Several factors may underlie the discrepancy between these seemingly opposing effects of avoidance behavior. First, our conception of avoidance learning is the discovery of instrumental actions that directly alter the probability of occurrence or intensity of an aversive outcome. Thus, clinical or laboratory studies investigating safety behaviors that have no actual causal influence over the aversive outcome may not engage the same psychological processes as our task. Moreover, tasks that explicitly instruct participants how to avoid the shock may circumvent a goal-directed avoidance learning process that may play a key role in mediating controllability effects (Amat et al. 2014). Finally, our studies in which AA has been shown to attenuate subsequent reactive threat responses have all involved at least a 24-h delay between avoidance learning and the test of Pavlovian responding (Boeke et al. 2017; Hartley et al. 2014). Thus, another possibility is that a time-dependent consolidation process may mediate the subsequent effects of avoidance learning. Future studies examining each of these factors will be required to better understand the circumstances under which avoidance behavior may promote or prevent the attenuation of subsequent fear expression.

Our yoked experimental design ensured an equivalent number of shocks across yoked pairs, while varying their respective degree of controllability. However, yoked designs pose challenges for equating all of the cognitive processes engaged during the task. Here, in order to ensure that participants in the yoked extinction group experienced a contiguous set of extinction trials, we altered the timing of shock administration for yoked extinction subjects whose active avoidance group counterpart received a shock late in the avoidance session. Nonetheless, our central finding was still evident in the yoked participant pairs for whom shock timing was not altered, suggesting that this violation of a strict yoking procedure did not drive our observed group differences. The demand for attention to the CS might be greater during performance of the avoidance response than during the yoked motor control task, in which participants only needed to attend to the red dots in order to respond correctly. However, tasks carrying greater attentional demands are typically associated with increased sympathetic arousal (Critchley 2002). As we observed lower SCR in the avoidance group than in yoked participants during the avoidance/extinction phase, our data do not support an interpretation that such attentional differences contributed to our effects.

A limitation of the present study is that the active avoidance and yoked extinction phases of the task differed not only in controllability of the shock, but also shock predictability. Once the subjects in the AA group learned the correct action, they could anticipate that they would not be shocked. Subjects in the yoked EXT group, however, had no way of knowing whether they would be shocked on a given trial. An alternative experimental design could control for predictability by allowing yoked extinction subjects to passively view the responses of the active avoidance subjects and learn their predictive properties. In such a design, the inference that observation of the instrumental action predicts shock omission would effectively constitute safety signal learning, as opposed to traditional extinction. Safety signal learning can effectively diminish Pavlovian threat responding, but previous research has shown that Pavlovian responses exhibit recovery when a safety signal is subsequently omitted (Lovibond et al. 2000; Wagner and Rescorla 1972). Thus, we do not expect that the pattern of responding observed in the yoked group would differ substantially even if the paradigm was modified to equate predictability across conditions, although future studies should test this directly.

In this study, we tested three hypotheses about the potential ways in which dopaminergic signaling might relate to behavior in our task, using blink rate as a putative index of dopamine function. Our exploratory analyses provided support for one of these hypotheses, with blink rate during conditioning (prior to the controllability manipulation) modulating the effects on control on Pavlovian responses the following day. For participants assigned to the active avoidance group, but not those exposed to yoked extinction, individuals with higher blink rates exhibited a greater attenuation of Pavlovian responding during the recovery test phase. Blink rate was not associated with the magnitude of conditioned responding during initial acquisition or during the avoidance/yoked extinction phase, or late in the retrieval phase for participants in either group. The selective emergence of this association during the recovery test phase, and only for participants who had experienced both uncontrollable Pavlovian fear acquisition as well as active instrumental control during the avoidance phase, suggests that the expression of individual active versus passive response biases, previously proposed to be modulated by dopamine (Cabib and Puglisi-Allegra 2012; Lloyd and Dayan 2016), might be most evident in contexts associated with heightened uncertainty about threat controllability. Another possibility is that this time-dependent effect reflects individual differences in the consolidation of active avoidance learning, which has previously been shown to be modulated by dopamine (Gozzani and Izquierdo 1976). Given the exploratory nature of the present analyses, future studies might be designed to corroborate this finding and directly test the underlying mechanisms.

Contrary to evidence in animal models suggesting that dopamine might facilitate avoidance learning (Darvas et al. 2011; Fibiger et al. 1974), we did not observe a relationship between blink rates and learning success for participants in the active avoidance condition. However, given the low proportion of non-learners, our study might not have had sufficient intersubject variability in learning or been adequately powered to detect such an effect. We also did not observe increases in blink rate during the avoidance phase as we hypothesized. Past studies relating dynamic changes in blink rate to task conditions examined fluctuations at the timescale of seconds (Rac-Lubashevsky et al. 2017; van Bochove et al. 2013), rather than averaging over a phase of several minutes as we did in our analysis. At our less granular timescale, individual blink rates were highly stable across all phases of the task for participants in both conditions, resembling a more trait-like individual difference measure. The origins of these individual differences in our participants are unclear. While blink rates might reflect tuning of central dopamine levels based on some aggregate degree of controllability of past experience, we have no life history data in our participants that could directly support this interpretation. Consistent with the idea that a trait-like behavioral bias toward proactive responding might be encoded via blink rate, past studies have found that individuals with a higher locus of control—an individual’s belief that environmental reinforcement can be influenced through their own actions—exhibit higher spontaneous blink rates (Declerck et al. 2006). However, our locus of control measure exhibited no relationship to participants’ blink rate or CRs. This absence of this relationship in our sample might stem from the use of a difference locus of control self-report measure, or the fact that our measure of eyeblink rate was obtained during aversive conditioning, as opposed to a spontaneous rate of blinking recorded during disengaged rest.

Persistent and debilitating fear is a core characteristic of multiple anxiety and stress-related disorders. Pavlovian aversive conditioning and extinction provide an experimental model for how these persistent fears are learned and how they can be regulated (Hartley and Phelps 2009; Milad and Quirk 2012). The primary behavioral treatments for anxiety disorders are exposure therapies, based on the principles of extinction learning, which desensitize fear associations through repeated passive exposure to a fear-eliciting stimulus (Foa and Kozak 1986). Extinction is thought to diminish fear by promoting the acquisition of a new safety association with the cue. However, the efficacy of such therapies is limited by the fact that the original extinguished fear memory often reemerges. Our finding that active avoidance prevented the reemergence of conditioned responding suggests that the development of therapeutic approaches that cultivate proactive instrumental strategies for coping in fearful situations may be more effective than extinction-based therapies at persistently attenuating fear (LeDoux and Gorman 2001; Van der Kolk 2006). Moreover, our results suggest that individual differences may modulate the efficacy of such therapies. Building upon this provisional finding, future studies might investigate whether pharmacological manipulation of dopamine might facilitate the degree to which active coping reduces fear recovery, suggesting a potential adjunct to behavioral therapies.

## Electronic supplementary material

ESM 1 (DOCX 16 kb)
