# Supplementary material for: Individual differences in blink rate modulate the effect of instrumental control on subsequent Pavlovian responding
Source: Psychopharmacology (Berl). 2018 Nov 1;236(1):87–97. doi: 10.1007/s00213-018-5082-6 (PMC6373194; doi:10.1007/s00213-018-5082-6)
Supplement: Supplementary file 1 — (DOCX 16 kb) [file 213_2018_5082_MOESM1_ESM.docx]

**SUPPLEMENTAL INFORMATION**

**Strict-Yoke Analysis**

Nine AA participants received shocks within the last 12 trials, despite having successfully avoided the shock on previous trials. Because shocking EXT participants midway through their yoked extinction phase would compromise their extinction learning, the timing of shock administration for these trials was shifted earlier in time for their nine EXT counterparts to the first eight trials, ensuring that all EXT participants experienced at least 12 contiguous unreinforced CS+ presentations during extinction. As this temporal shift violates a strict yoking of EXT and AA participants, all SCR analyses reported in the main text are also repeated here in a “strict-yoke” i.e., master-yoke pairs for whom the timing of shocks was the same) sub-sample of the remaining 15 yoked AA-EXT pairs, who experienced identical timing of shocks.

In the strict yoke sample (N = 15 per group), the group x Block ANOVA on CRs again revealed a main effect of block (F(1.65,46.24) = 3.81, p = .04) and no effect of group (F(1,28) = .72, p =.4). However, the group x block interaction failed to reach significance (F(1.65,46.24) = .3).

Again, both groups acquired a CR (AA: t(14) =4.62, p = .0004; EXT: t(14) = 4.96, p = .0002), and CR magnitude in late acquisition did not differ (t(28) = .06, p = .96). Conditioned responding was marginally lower in late AA/EXT than in acquisition for both groups (AA: t(14) = 1.76, p = .1; EXT: t(14) = 1.92, p = .08), and CRs did not differ between groups during AA/EXT (t(28) = .01, p = .99). During early retrieval, AA subjects showed lower CRs than EXT subjects (t(28) = 2.08, p = .046). The CR of the EXT group during early retrieval was significantly greater than zero (t(14) = 5.9, p =00004) and marginally increased from late EXT (t(14) = 1.86, p = .08), whereas the CR of the AA group during early retrieval was marginally greater than zero (t(14) = 1.95, p = .07) and not significantly different from late AA t(14) = .04, p = .97). Again, the AA group showed a decreased CR during retrieval compared to acquisition (t(14) = 2.31, p = .04), while the EXT group did not (t(14) = .15, p = .88).

Thus, the results in the strict yoke sample follow the same pattern as the full sample. Though the block x group interaction is no longer significant in this limited sub-sample, likely due to the diminished sample size, the key finding of reduced CR in AA subjects compared to EXT subjects during retrieval remains significant.
